# Desmoplastic histological subtype of ameloblastoma in 16 dogs

**DOI:** 10.3389/fvets.2024.1362237

**Published:** 2024-04-04

**Authors:** Kristina Feigin, Cynthia Bell

**Affiliations:** ^1^Veterinary Dental Services, Boxborough, MA, United States; ^2^Specialty Oral Pathology for Animals, Geneseo, IL, United States

**Keywords:** desmoplastic ameloblastoma, histopathology, dog, odontogenic, neoplasia, radiology, dentistry, veterinary

## Abstract

Ameloblastoma in dogs most often has a highly conserved acanthomatous cell morphology and is recognized as canine acanthomatous ameloblastoma (CAA) while conventional ameloblastoma (CA) makes up a smaller yet morphologically diverse group of epithelial odontogenic tumors. In humans, a rare desmoplastic histological subtype has distinctive clinical, radiological, and microscopic features. Desmoplastic ameloblastoma (DA) has not previously been described in dogs, although it has been rarely referenced in the veterinary literature. This is the first thorough description of a case series of DA in dogs and describes clinical presentation, diagnostic imaging findings, histopathological features for diagnosis, and treatment outcome. Clinically, DA most often presents as a mass or swelling in the rostral mandible or maxilla of middle age to older dogs. On diagnostic imaging, the lesion has a radiolucent or mixed pattern with well-defined borders and variable loculation. As a solid, fibrous tumor with obscured odontogenic epithelium, DA is challenging to diagnose histologically and can mimic several other oral tumors, both benign and malignant. As an ameloblastoma, the biological behavior of DA is locally destructive yet benign and prognosis is favorable following surgical excision.

## Introduction

Ameloblastoma in dogs most often has an acanthomatous histologic subtype and Canine acanthomatous ameloblastoma (CAA) is recognized as an entity that is distinct from other ameloblastomas in dogs (1–3). CAA has been studied extensively and is widely recognized by clinicians and pathologists (4–6). The smaller proportion of ameloblastomas in dogs that are not CAA have diverse histologic morphologies and represent a heterogeneous group of epithelial odontogenic tumors that have not been well-characterized and are often grouped in the category of Ameloblastoma or Conventional ameloblastoma (2, 7). The histological variants of Conventional ameloblastoma are better characterized in humans than dogs and include plexiform, follicular, acanthomatous, and desmoplastic among others (8). The veterinary literature reports one desmoplastic ameloblastoma in a dog without any specific details of the case (9).

The desmoplastic subtype of ameloblastoma can be difficult to recognize since classic histomorphological features of odontogenic epithelium are often lacking or difficult to recognize (10, 11). Misdiagnosis risks erroneous prognostication and inappropriate or delayed treatment for canine patients. In humans, DA is reported most often in the anterior and premolar regions in middle aged adults (10–12). Radiographically, DA appears as a mixed radiolucent and radiopaque swelling, often resembling a benign fibro-osseous lesion (10–12). This manuscript provides clinical, radiological, and histopathological data for 16 odontogenic tumors in dogs that were recognized as having features compatible with the diagnosis of DA as it is applied in humans.

## Materials and methods

Cases were identified in a search of oral biopsy submissions from dogs that were submitted to a single pathology laboratory in the timeframe from June 2020 to July 2023 (38 months). All canine cases submitted to the lab during this timeframe were evaluated in order to determine the relative frequency of desmoplastic ameloblastoma (DA), conventional ameloblastoma, and canine acanthomatous ameloblastoma. Detailed clinical, radiological, and histological features were evaluated only for cases that had features compatible with desmoplastic ameloblastoma as described in humans (8, 10–14).

Inclusion as desmoplastic ameloblastoma required a gingival or intra-osseous mass in the tooth-bearing region of the mandible or maxilla and histological confirmation of odontogenic epithelium with densely collagenized stroma (i.e., desmoplastic pattern). The desmoplastic pattern must account for >50% of the mass. It was necessary for epithelium to be widespread throughout the mass. Tumors with only localized odontogenic epithelium were excluded. Both abundance and histological morphology of epithelium could vary, although it was necessary for some of the epithelium to be identifiable as odontogenic. Small polygonal epithelial cells arranged in packets (resembling odontogenic epithelial rests) and thin branching cords (resembling epithelium of the dental lamina) were interpreted as confirmation of odontogenic epithelium. Finally, cases were excluded if neoplastic tissue infiltrated oral mucosa.

Signalment, clinical history, and clinical findings were determined based on medical records and direct communication with veterinarians. The following parameters were recorded: age, sex, breed, neutering status, mass size, previous histopathological diagnosis, surgical intent and treatment, and any evidence of recurrence and follow up. Tumor size was determined by the description in the mass in the clinical record, by size provided on the biopsy submission form, or by direct measurement of the formalin-fixed gross lesion. Treatment was categorized as either resection/ectomy of the mass or curettage/enucleation. Local lymph node evaluation and aspiration and as well as any staging for metastasis was noted. The lesion was considered rostral if it was centered on canine and incisor teeth and premolar if it was centered on the premolar teeth.

Intra-oral dental radiographs and/or CT/CBCT studies were reviewed by a board-certified veterinary dentist. The following parameters were evaluated: location of the lesions, definition of margins (well vs. ill-defined), locularity (unilocular vs. multilocular), presence of cortication, tooth displacement and tooth resorption. The lesion was considered unilocular if presented as a single mixed or radiolucent mass, and multilocular when multiple lucent areas appeared divided. No locularity was also noted if evident. The internal appearance of the lesion was classified into one of three basic categories: totally radiolucent, totally radiopaque, or mixed (mixed radiolucent and radiopaque). DA was considered radiolucent when presented as radiolucent shade and mixed when it was radiolucent/radiopaque. The borders were evaluated as well-defined if the lesions were clearly demarcated or ill-defined when the lesions had an indistinct boundary. The former can be corticated and non-corticated. Corticated margin is a thin, fairly uniform radiopaque line of reactive bone at the periphery of the lesions. Presence or absence of cortical expansion was noted.

All tissue samples were formalin-fixed and processed routinely, embedded in paraffin, sectioned and stained with hematoxylin and eosin. Tissues requiring decalcification were treated with hydrochloric acid (Decal Stat, StatLab) prior to histological processing. For immunohistochemistry (IHC), paraffin embedded, 5 μm-thick sections were deparaffinized and rehydrated, and antigen retrieval was performed (Supplementary Table S1). Tissue sections of the neoplasm (cases 5, 13, 14, and 16) were immunohistochemically labeled for vimentin V9, cytokeratin AE1/AE3/PCK26, and cytokeratin MNF116 (Supplementary Table S1). IHC was performed on a BenchMark Ultra system using an ultraView Universal DAB Detection Kit with hematoxylin counterstain (Supplementary Table S1). All histopathological interpretation was performed by a board-certified veterinary pathologist.

The recorded microscopic parameters included morphology of neoplastic epithelial cells, organization of stromal tissue (cellular vs. fibrous), keratinization of neoplastic epithelium, presence of cysts formed by the neoplastic epithelium, presence and abundance of osseous matrix/dentinoid, and mitotic count (as figures per 10 high-power fields, 2.37 mm^2^). Osseous matrix/dentinoid was quantified as none, mild (present in <25% of tumor evaluated), moderate (25–50%), or abundant (>50%). Microscopic margin evaluation was performed and the narrowest histological margin was recorded in centimeters.

## Results

### Clinical findings and history

Data for each canine patient is presented in Table 1. The female to male ratio was 1:1 and the age ranged from 5 to 13 years with a mean of 9.5 years. No predominant breed was noted and patients varied from mixed, small, medium, and large breed; including brachycephalic and dolichocephalic skull conformation. In the specified timeframe, 17,377 oral pathology submissions from dogs had been received and evaluated, including 1,849 cases that were diagnosed as ameloblastoma. Of the ameloblastoma cases, 1,510 (81.7%) were canine acanthomatous ameloblastoma (CAA), 187 (10.1%) were conventional ameloblastoma (CA), 62 (3.3%) were amyloid-producing ameloblastoma, 16 (0.9%) were desmoplastic ameloblastoma, and 74 (4.0%) could not be specified as CAA or other type of ameloblastoma. Keratinizing squamous cells can be a prominent feature of both CA and amyloid-producing ameloblastoma, therefore, keratinizing ameloblastoma was not recognized as a separate category.

**Table 1:** Clinical features of desmoplastic ameloblastoma in dogs.

**Case #**	**Breed**	**Age (years)**	**Sex**	**Duration (months)**	**Tumor size**	**Mitotic count^a^**	**Histological margin**	**Tumor recurrence**	**Deceased**	**Follow up (months)**
1	Dachshund mix	5.5	MN	4	4.0 × 3.8 × 2.3 cm	< 1	Incomplete	Yes^*^	No	49
2	Great Dane	8	FS	36	6.5 × 5 × 4 cm	< 1	Incomplete	Yes/no^**^	Yes	34
3	Cocker spaniel	11	FS	7	2.7 × 2.7 × 1.6 cm	1	0.1 cm	No	No	27
4	Bassett hound	9	M	1	2.3 × 1.9 × 1.8 cm	< 1	0.6 cm	No	No	24
5	Pug	13	MN	2.5	5.0 × 3.7 × 3.2 cm	< 1	1.0 cm	No	No	NA
6	Mixed breed	9	FS	2	2.3 × 2.2 × 2.0 cm	< 1	0.7 cm	No	No	6
7	Australian shepherd	12	FS	3	2.7 × 2.5 × 2.4 cm	9	1.0 cm	No	Yes	14
8	Mixed breed	5	MN	NA	3.5 × 3.0 × 2.6 cm	2	0.1 cm	No	No	8
9	Pitbull mix	11	MN	NA	1.5 × 2.0 × 1.5 cm	< 1	0.2 cm	No	No	6
10	Golden retriever	5	MN	2.5	3.5 × 2.0 × 3.0 cm	< 1	0.4 cm	No	No	10
11	Shetland sheepdog	10.7	FS	7	4 × 5 × 2 cm	< 1	0.5 cm	No	No	6
12	Retriever mix	12	FS	1	1.7 × 1.0 × 1.3 cm	< 1	0.1 cm	No	No	4
13	Labrador retriever	8	M	1.5	2.7 × 2.4 × 2.0 cm	< 1	0.8 cm	No	No	3
14	Pitbull	11	MN	0.75	3.0 × 3.3 × 2.0 cm	11	0.6 cm	No	No	3
15	Wheaton terrier	10	FS	Unknown	4.4 × 4.0 × 3.2 cm	5	incomplete	No	No	2
16	Hound mix	11	FS	2	2 × 2.5 × 2 cm	< 1	0.2 cm	No	No	5

^a^Mitotic count is reported as figures per 10 high power fields (2.37 mm^2^).

^*^This patient has had 4 surgical debulking procedures but no mandibulectomy for curative intent.

^**^Regrowth followed initial debulking of the tumor, although no recurrence was reported subsequent to more aggressive surgery.

NA, this patient was lost to follow up.

A histopathological diagnosis based on previous biopsy had been given for 14 of 16 cases. Four patients had a diagnosis of odontogenic neoplasm that was not further specified. Four patients had a diagnosis of peripheral odontogenic fibroma. Three patients had a diagnosis of fibrous or spindle cell proliferation with differentials including peripheral odontogenic fibroma, fibrosarcoma, or other spindle cell neoplasm. Three patients had a diagnosis of epithelial neoplasm with differentials including squamous cell carcinoma, ameloblastoma, or other epithelial tumor.

**Figure 1:**
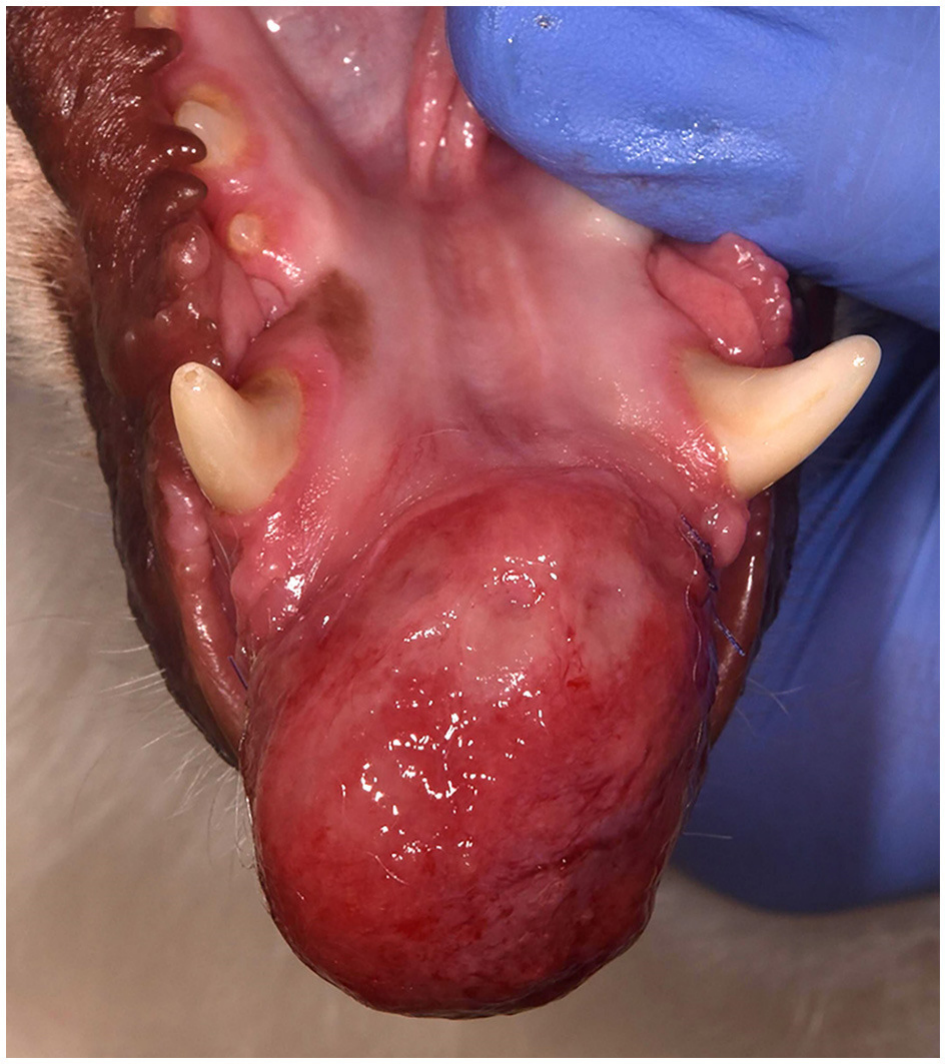
Clinical image of a large rostral mandibular DA (case #7). The mandibular incisors are missing due to previous extraction at the time of the incisional biopsy.

**Figure 2:**
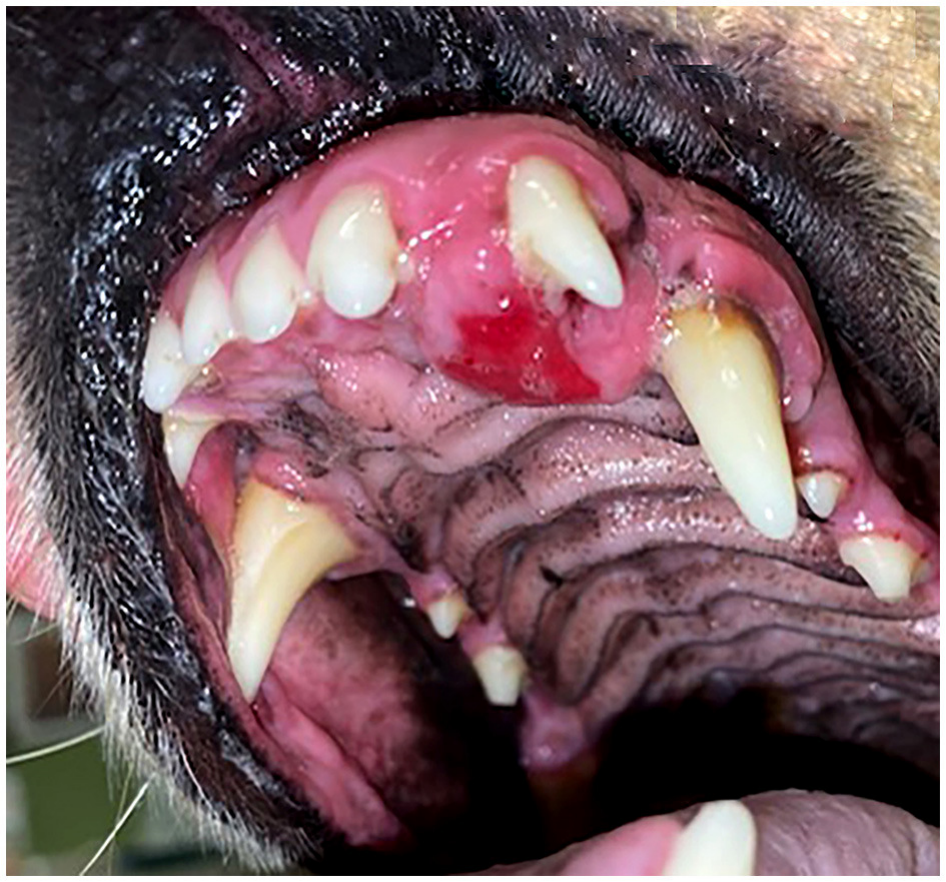
Clinical image of a left rostral maxillary DA (case #10) that has caused distal displacement of tooth 203.

**Figure 3:**
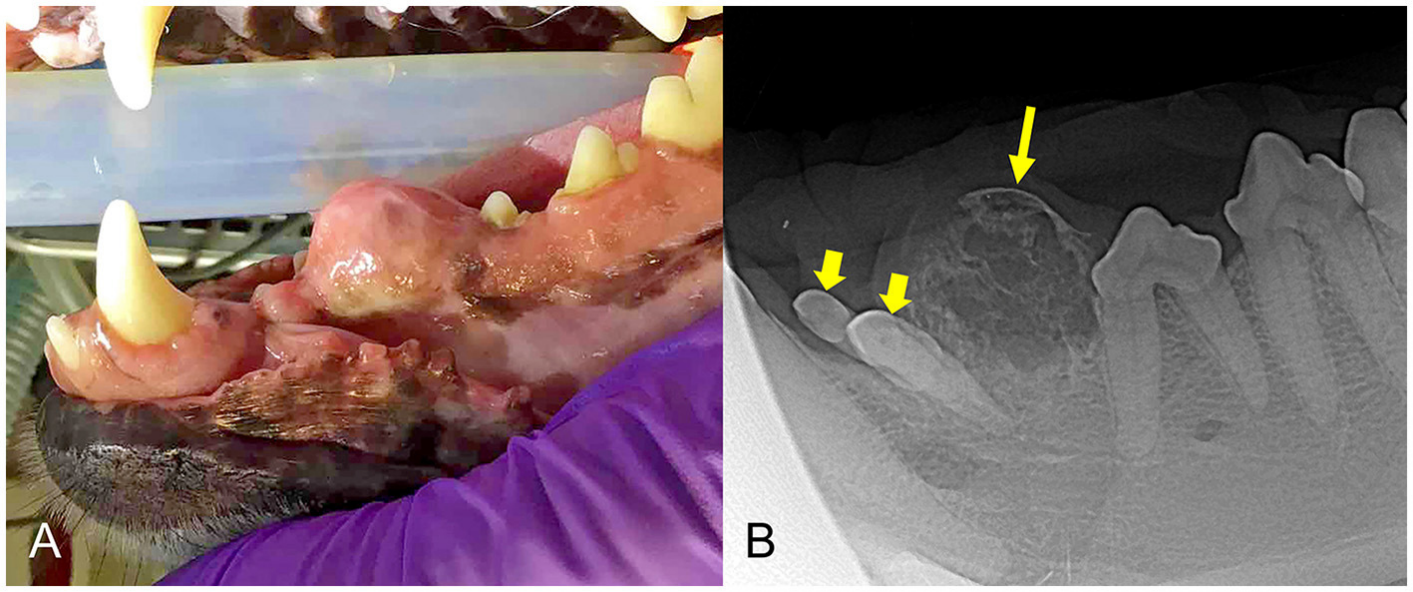
**(A)** Clinical image of a non-ulcerated, well-defined, left mandibular DA (case #4) in the area of premolars 306–307. **(B)** Lateral dental radiograph of the left mandible (case #4). A well-defined, round mass with mixed density has evidence of cortication (long arrow) and teeth 305 and 306 are displaced (short arrows).

**Figure 4:**
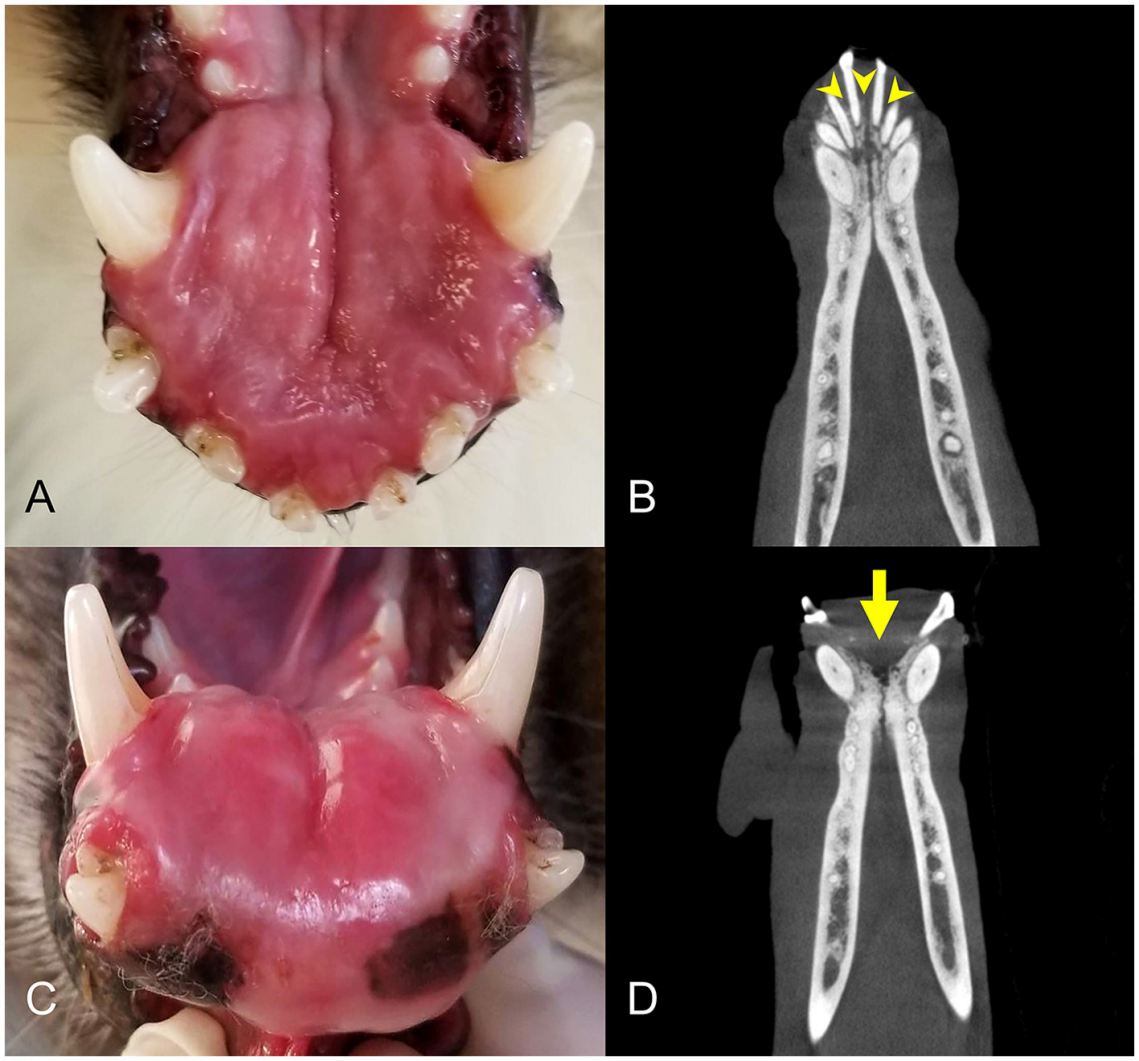
Clinical photographs and diagnostic images taken 1 year apart of a rostral mandibular DA (case #11). **(A)** Photo at initial presentation, appearing as a rostral mandibular swelling. **(B)** Dorsal plane CBCT view at initial presentation. There is mild loss of mandibular bone and rostral displacement of the mandibular incisors (arrowheads). **(C)** Clinical photograph of the intraoral mass taken 1 year later, showing progression of the rostral mandibular swelling. **(D)** Dorsal plane CBCT view 1 year later demonstrating more extensive osteolysis of the rostral mandible (arrow).

Patients with DA frequently presented with a rostrally placed mass/swelling that was noted to be present anywhere from 3 weeks to 3 years. Clinically, this tumor had predilection for occurrence in the rostral maxilla and mandible (75%) with fewer cases in the premolar region (25%). There were no DA centered on the molar teeth. There was almost equal distribution between the maxilla and mandible. Mass size ranged from 1.7 × 1.0 × 1.3 cm to 6.5 × 5 × 4 cm and the average size was 29.0 cm^3^. The tumor was commonly described as a raised, firm gingival mass and was occasionally ulcerated and frequently caused tooth displacement. Three cases had missing teeth in the area of the mass, so displacement could not be confirmed or denied (Figure 1). All of the cases that had teeth in the area of the mass had tooth displacement (Figures 2–4).

### Diagnostic imaging

**Figure 5:**
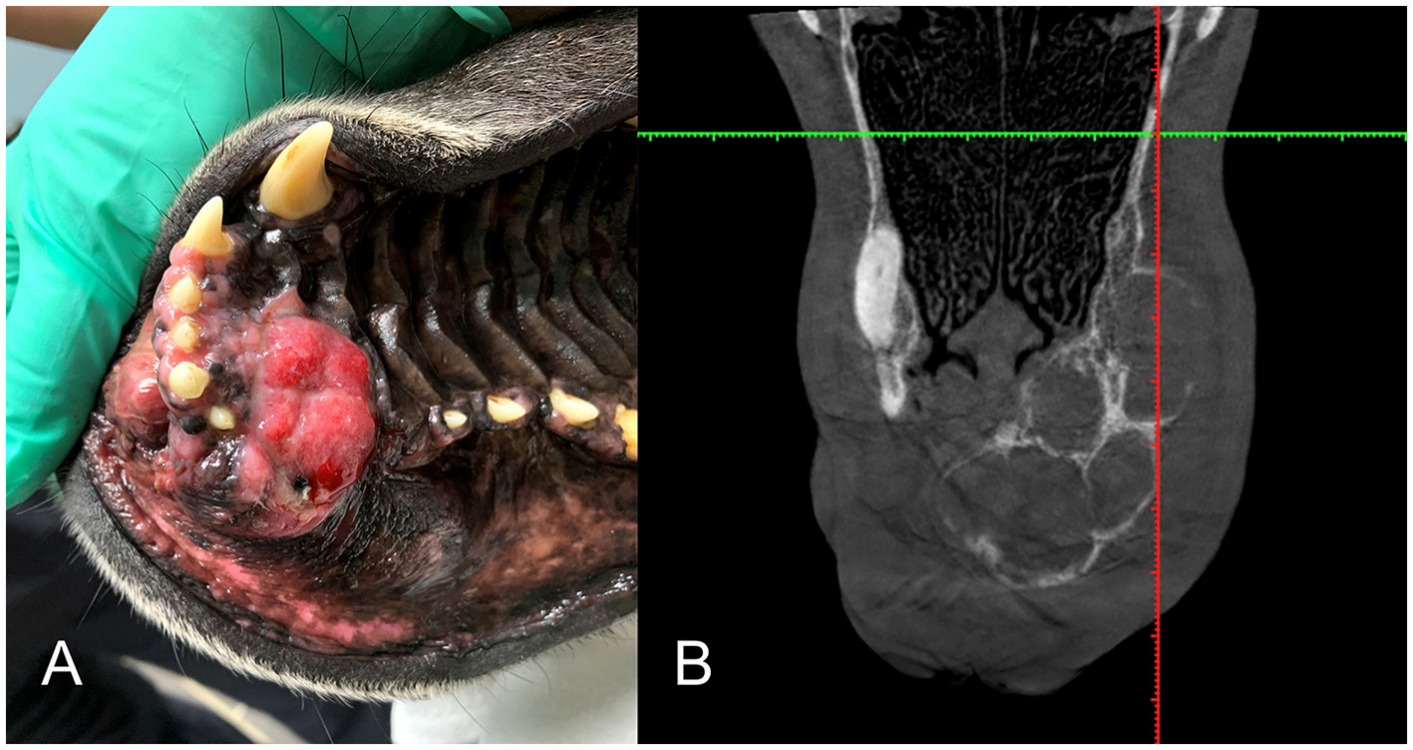
**(A)** Intraoral clinical view of a right maxillary DA (case #2). **(B)** Dorsal plane Conventional Tomography view (case #2) is an example of the multilocular, soap-bubble type lesion.

Diagnostic imaging was available for all 16 patients and results of radiological evaluation are in Table 2. Dental radiographs were provided for 13 (81.2%) patients and 11 (68.7%) patients had CT or CBCT. A radiolucent lesion was present in seven cases (43.7%), and when bone production was observed, the pattern was mixed lytic/proliferative in nine cases (56.2%) (Figure 3B). In some cases, radiopaque flecks were scattered around the radiolucent region. The tumor presented radiologically as a unilocular lesion in 7 (43.7%) cases (Figure 3B), a multilocular radiolucency in 3 (18.7%) cases (Figure 5B), and no locularity was noted in 6 (37.5%) cases (Figure 4D). The tumor frequently resulted in displacement of the affected teeth (Figures 3B, 4B), and tooth resorption was not observed in any case. Radiographic borders were well-defined for 13 (81.7%) tumors and 3 (18.7%) had ill-defined borders. Six (37.5%) tumors had evidence of expansion with cortication (Figure 3B). One patient had conventional CT with contrast and the lesion was noted to be enhancing.

**Table 2:** Diagnostic imaging findings for desmoplastic ameloblastoma in 16 dogs.

**Case**	Location^*^	**Borders**	**Locularity**	**Radiological pattern**	**Cortication**	**Tooth resorption**	**Tooth displacement**
1	Right mandible, 404–408	Well-defined	Multi	Mixed	No	No	Yes
2	Rostral maxilla, 201–105	Well-defined	Multi	Mixed	Yes	No	N/a
3	Left maxilla, 204–207	Well-defined	Uni	Mixed	Yes	No	N/a
4	Left mandible, 305–307	Well-defined	Uni	Mixed	Yes	No	Yes
5	Left maxilla, 202–206	Well-defined	Multi	Mixed	Yes	No	Yes
6	Rostral mandibles, 301–403	Ill-defined	No	mixed	No	No	N/a
7	Right mandible, 403–404	Well-defined	Uni	Lucent	No	No	Yes
8	Left maxilla, 207–208	Well-defined	Uni	Lucent	Yes	No	Yes
9	Rostral maxilla, 102–201	Well-defined	No	Mixed	No	No	Yes
10	Left maxilla, 201–204	Well-defined	No	Lucent	Yes	No	Yes
11	Rostral mandibles, 304–404	Well-defined	No	Lucent	No	No	Yes
12	Right mandible, 404	Well-defined	No	Lucent	No	No	Yes
13	Rostral mandibles, 303–402	Ill-defined	Uni	Mixed	No	No	Yes
14	Rostral mandibles, 303–404	Well-defined	Uni	Lucent	No	No	Yes
15	Rostral mandibles, 305–406	Well-defined	Uni	Lucent	No	No	Yes
16	Left maxilla, 202–203	Ill-defined	No	Mixed	Yes	No	Yes

^*^Tooth number is according to the modified triadan system.

### Histopathology

**Figure 6:**
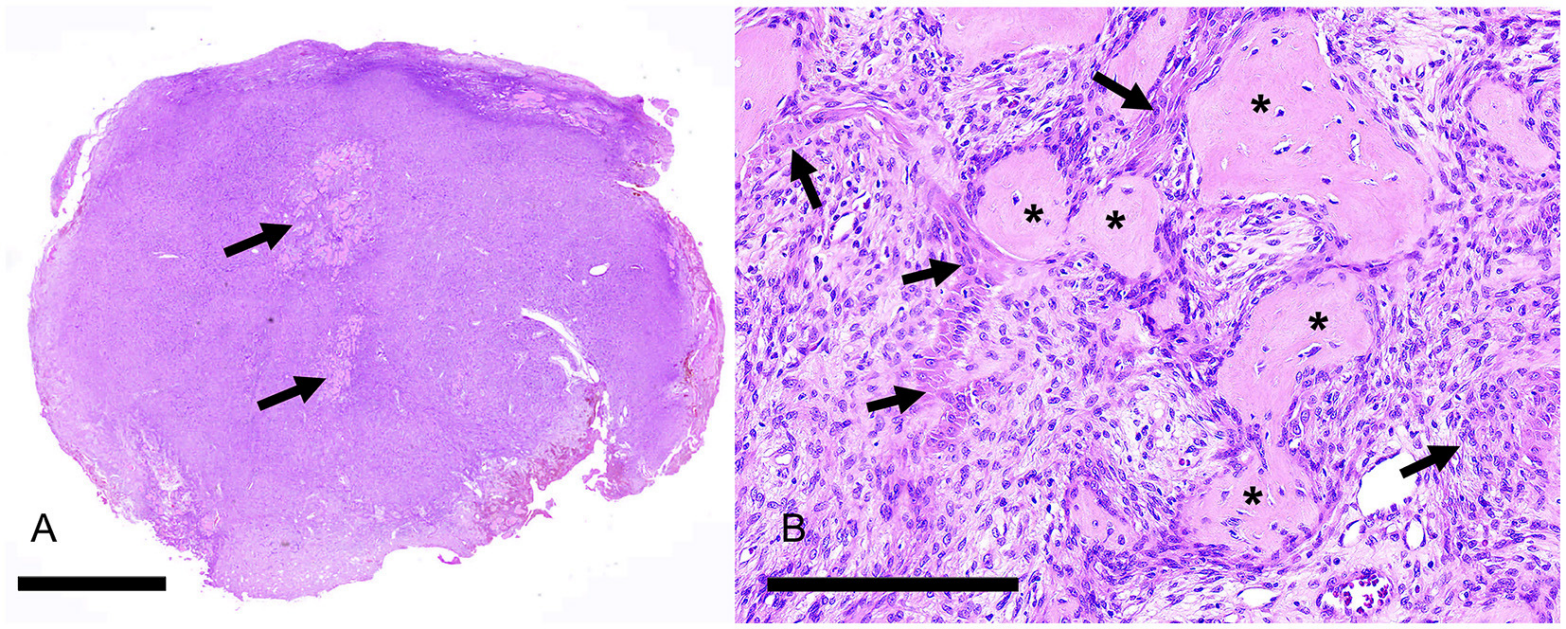
Photomicrographs of a rostral maxillary desmoplastic ameloblastoma (case #2), HE stain. **(A)** Although this tumor had a multilocular appearance on CT (see Figure 5B), the mass was solid rather than cystic and had multifocal areas of osseous matrix (arrows). Bar = 4 mm. **(B)** At higher magnification, the neoplasm has some distinct cords of epithelium (arrows) within a cellular background where epithelial cells are not easily differentiated from mesenchymal stromal cells. The osseous islands (asterisks) are sometimes intimately associated with the epithelium, almost appearing as if the osteoid is being produced by polygonal cells. Bar = 200 um.

**Figure 7:**
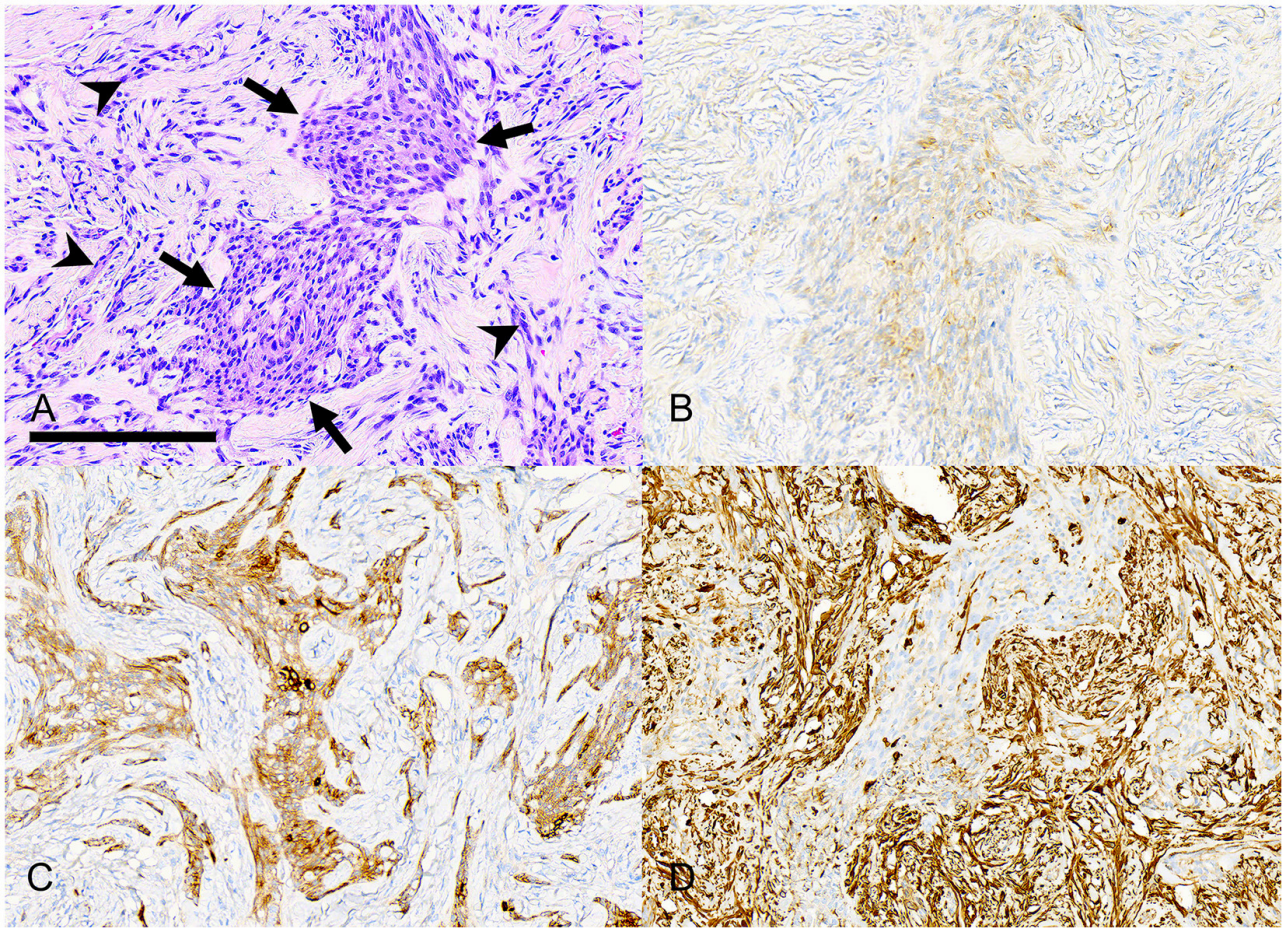
Photomicrographs of a rostral maxillary desmoplastic ameloblastoma (case #5). **(A)** An irregular island of neoplastic odontogenic epithelium (arrows) has polygonal to stellate to plump spindle cells. The supporting collagenous stroma has many smaller, distorted cords and nests of similar cells (arrowheads). Bar = 200 μm, HE stain. **(B)** The neoplastic epithelial cells have weak immunolabeling for pankeratin AE1/AE3/PCK26. **(C)** The neoplastic epithelial cells have stronger immunolabeling for pankeratin MNF116. **(D)** The neoplastic epithelial cells are negative for vimentin, although there is strong positive immunolabeling of stromal spindle cells.

**Figure 8:**
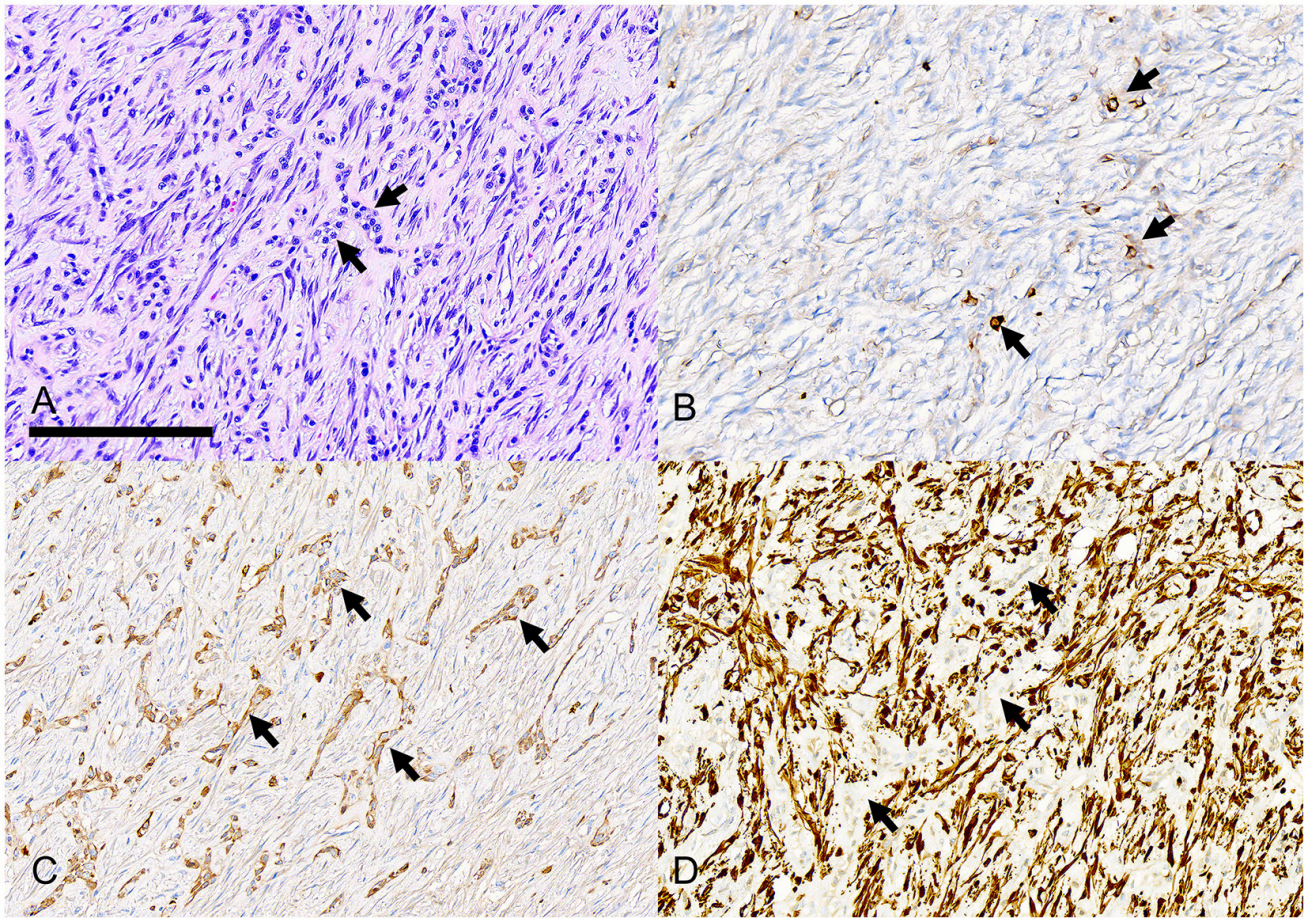
Photomicrographs of a rostral mandibular desmoplastic ameloblastoma (case #14). **(A)** Short interrupted cords of neoplastic odontogenic epithelium (arrows) are difficult to differentiate from a moderately cellular fibrous stroma with plump spindle cells and pink-staining collagen fibers. Bar = 200 um, HE stain. **(B)** Only rare individual neoplastic epithelial cells have positive immunolabeling for pankeratin AE1/AE3/PCK26. **(C)** Several cords and nests of neoplastic epithelial cells have positive immunolabeling for pankeratin MNF116. **(D)** Stromal spindle cells have strong positive immunolabeling for vimentin although the cords of neoplastic epithelium (arrows) are negative for vimentin.

Individual tumors were solid (Figure 6) with variable amounts of epithelium and epithelial cell morphology ranging from polygonal to stellate to spindle. The neoplastic epithelium formed nests, islands, and cords (Figure 7). The broadest islands of neoplastic epithelium often had larger squamous cells with 11 (68.8%) tumors having small foci of squamous differentiation. The neoplastic epithelium formed one or more cysts in 6 (37.5%) tumors. Epithelial cells with spindle or indefinite shape were streaming or isolated within the collagenous stroma; as a result, the epithelial cells were often difficult to differentiate from stromal cells (Figure 8). Immunohistochemistry for pankeratins was useful for identifying epithelial cells that were morphologically indistinct (Figures 7, 8). Thus, neoplastic odontogenic epithelium in desmoplastic ameloblastoma is frequently distorted or hidden within a desmoplastic background of spindle cells and collagen.

The stromal tissue was also variable with respect to cellularity and density with 12 (75%) tumors having a poorly cellular fibrous stroma and 4 (25%) tumors having more prominent stromal cells. Osseous matrix was present in 12 (75%) tumors (Figure 5) and did not depend on the presence of abundant collagen since tumors without osseous matrix were split equally between having a fibrous or cellular stroma. The quantity of osseous matrix was most often mild (7/12 tumors, 58.3%) and less often moderate (3/12 tumors, 25%) or abundant (2/12 tumors, 16.7%).

Despite lack of histological organization within DA lesions, the mitotic counts were low and nuclear pleomorphism was consistently mild to moderate. Twelve (75%) tumors had 1 mitotic figure or fewer per 10 high-power fields (hpf, 2.37 mm^2^) and the maximum mitotic count was 11 figures per 10 hpf. Soft tissue borders of DA were well-defined and the neoplasm was often pseudo-encapsulated due to compression of adjacent soft tissues. The neoplastic tissue was mildly infiltrative within bone, resulting in a narrow zone of transition between the neoplasm and surrounding normal bone.

### Treatment and follow-up

In 13 of 16 patients, the tumor was completely excised with margins ranging from 0.1 to 1.0 cm; none of these have recurred as of the time of writing with follow up ranging from 2 to 27 months. One patient (case 1) has had a total of 4 debulking surgeries and has survived over 4 years and is reported to be in good health. Another tumor (case 2) recurred after debulking and persisted for 3 years at which time aggressive debulking did not achieve histologically “clean” margins but the patient survived another 34 months before death for an unrelated cause. One dog in the study (case 7) was euthanized due to sudden onset of respiratory distress, the cause of which was not determined. This patient had survived 14 months without recurrence of the oral mass. One dog was lost to follow-up (case 5). All others are alive at the time of last recheck, ranging from 2 to 49 months post-surgery.

Clinical staging procedures were performed for only a few patients. Five (31.2%) patients had normal preoperative chest radiographs and 11 (68.7%) did not have chest radiographs taken prior to surgery. One patient (6.25%, case #1) was noted to have had slight enlargement of the ipsilateral mandibular lymph node, 11 patients (68.7%) had reportedly normal mandibular lymph nodes based on palpation, and four cases (25%) had no written notes about the state of the mandibular lymph nodes. None of the evaluated medical records indicated that lymph nodes had been aspirated for evaluation.

## Discussion

Desmoplasia refers to proliferation of fibrous connective tissue. Desmoplastic types of various epithelial tumors are characterized by having a prominent fibrous stroma. Desmoplasia is most often a feature of malignant epithelial neoplasms (e.g., squamous cell carcinoma, mammary adenocarcinoma) and may have prognostic significance (15). Although less common, desmoplastic variants of benign epithelial tumors occur, including tumors of hair follicle epithelium (16). Desmoplastic ameloblastoma is an example of stromal desmoplasia in a benign epithelial tumor. Sclerosing odontogenic carcinoma is another neoplasm of odontogenic epithelium with desmoplastic stroma, which was recently added to the WHO classification for humans (8, 17). There are only a few cases of this entity reported and it is not considered pertinent to this discussion since the sclerosing odontogenic carcinoma is described as highly aggressive with perineural infiltration and invasion into adjacent soft tissues (18).

### Desmoplastic ameloblastoma in humans

A discussion of Desmoplastic ameloblastoma (DA) in humans is warranted since this neoplasm has not been previously described in dogs and because the clinical, radiological, and histological features of the canine cases presented here are remarkably similar to DA in humans.

The desmoplastic ameloblastoma (DA) in humans was first described in the 1980s (12, 13). Historically, DA was recognized as a distinct and separate type of ameloblastoma; however, since 2017, the World Health Organization (WHO) classification of odontogenic tumors in humans includes the desmoplastic histological subtype within the larger ameloblastoma group (8, 17). This group has variably been called conventional ameloblastoma, classic intraosseous ameloblastoma, and solid/multicystic ameloblastoma (8, 17). The justification for including of DA into the larger group was based on studies that did not prove a difference in biological behavior or prognosis despite DA having radiological and histological features that are unique from other conventional ameloblastoma (10, 19). Furthermore, individual ameloblastomas rarely have only one histological pattern and the occurrence of “hybrid” ameloblastomas (having a desmoplastic pattern intermixed with plexiform or follicular organization) further suggests that DA is a subtype of conventional ameloblastoma (12). DA in humans is rare, accounting for ~4 to 13% of ameloblastomas (10).

In humans, DA is reported most often in the anterior and premolar regions in middle aged adults with nearly equal sex distribution (10–12). The most common presentation is as a painless swelling with buccal extension (10). Mandibular tumors are slightly more common than maxillary (10, 11). Radiographically, tumor borders are usually poorly defined and the lesion appears as a mixed radiolucent and radiopaque swelling, often with root displacement (10–12, 20, 21). Approximately 50% are multilocular on diagnostic imaging (10). The multilocular type with septa is sometimes referred to as soap-bubble or honeycomb shaped (20, 21).

Histologically, most DA tumors have cords of odontogenic epithelium, although the follicular pattern that is typical of ameloblastoma in humans is generally absent (10–12). The epithelium can be distorted or “swirled” (10, 11). Stellate reticulum-like cells and ameloblastic cells are largely absent; the peripheral cell layer is generally attenuated and cuboidal rather than columnar with reverse nuclear polarity (10–12). The supporting stromal tissue has extensive collagenization and osteoplasia may be present (10–12, 14). The stroma immediately surrounding islands of odontogenic epithelium may have a loose, myxoid appearance (10, 12). Squamous metaplasia and foci of keratinization may be seen but are rarely a prominent feature (12). Small central cystic or duct-like spaces can be seen within epithelial islands (12).

In humans, ameloblastoma is diagnosed by biopsy and imaging, preferably a CT scan, and aggressive en bloc resection is the usual and recommended treatment (22). In humans, neither chemotherapy nor radiotherapy are routinely used for management of ameloblastoma (22). Rate of recurrence following surgical resection of DA is 3.1% compared to 21.1% recurrence risk following enucleation and the average period until recurrence is 36.9 months (10). According to one study, the most important variables that influence recurrence of ameloblastoma are time elapsed from treatment, type of surgical treatment, tumor size, and radiographic presentation (23).

Mutations identified in humans with ameloblastomas have affected genes of the MAPK pathway; specifically, BRAFV600E mutations are predominantly in mandibular ameloblastoma while SMO mutations are mostly found in maxillary tumors (22). Treatment with BRAF inhibitors can be effective in reducing ameloblastoma size and facilitate excision (22).

### Desmoplastic ameloblastoma in dogs

The canine tumors in this study are highly analogous to DA in humans in all of the related parameters including location, presentation, age, distribution within the jaw, radiological findings, histological findings, and biological behavior. Data from this study, based on biopsy submissions that are predominantly from veterinary dental specialists, suggests that CAA is ~8-fold more common than CA and fewer than 10% of CA are the desmoplastic variant. Thus, DA is a rare odontogenic neoplasm, accounting for just < 1% of all ameloblastoma and the ratio of CAA to DA is approximately 100:1. In comparison, DA accounts for up to 14% of ameloblastomas in humans (10). The difference between species is likely explained by CAA, which is unique to and remarkably common in dogs.

Based on evaluation of these canine cases, DA has no sex or breed predilection. Middle-aged to older dogs were affected, which is typical for neoplasia in dogs in general. The most frequent location of desmoplastic ameloblastoma in dogs appears to be the rostral mandible or maxilla, often causing tooth displacement, which is similar in humans (10–12). Clinically, the tumors in dogs were described by the submitting veterinarian as gingival or mucosal, although there was invariably bone involvement of the mandible or maxilla. The mass or swelling was occasionally ulcerated, particularly when the location of the mass promoted occlusal trauma. The largest mass (6.5 × 5 × 4 cm, case 2) had been present for 3 years and this Great Dane patient survived another 34 months without regrowth following marginal surgical excision. Therefore, the rate of growth is likely slow despite the ability of these tumors to become quite large.

One might expect DA to present as a smaller solid mass compared to CA, which often has multiple cysts contributing to tumor size (7). In humans, DA may be smaller on average than other types of ameloblastoma (10). However, the average size of tumors in this study was 29.0 cm^3^ and this is only minimally less than the average size of 31.2 cm^3^ for CA in dogs from a previous study (7).

Accurate radiologic examination and its relationship with the surrounding anatomic structures is considered essential preoperatively. Both the dental radiographs and, when available, 3D imaging (CT/CBCT) frequently showed predominantly radiolucent to mixed radiolucent/radiopaque lesions for DA in dogs. In some cases, radiopaque flecks were scattered around the radiolucent region. This correlates with the presence of an osseous matrix seen in 75% of the tumors in this study. Radiolucent interior is suggestive of an osteolytic process inside the jaw, which is a process of progressive destruction of bony tissue radiographically characterized as progressive radiolucency.

In humans, the mixed radiographic appearance of DA is also attributed to osteoplasia (14). The lesions presented radiologically as mostly unilocular or without locularity. Smaller number of cases had multilocular radiolucency. The lesions in this study frequently resulted in displacement of the affected teeth, without any signs of root resorption. CT evaluation in humans with DA also showed frequent displacement of teeth, however they also noted root resorption in 25% of the reported cases (10). A little more than a third of the cases had peripheral corticated border which indicates a slow-growing pattern. Well-defined corticated radiolucencies in the jaws of dogs are often odontogenic cysts and benign odontogenic tumors as they are slow growing and allow the bone surrounding them to remodel (7).

Other radiographic conclusions in this study include well-defined borders for the majority of the cases (13/16, 81.7%) which is similar to humans where almost all of the reported cases appear to have well-defined borders both on CT and MRI imaging (10, 21). This feature can be very helpful and increase the degree of confidence for the clinician for border evaluation and measurement of the anticipated surgical margins. Anecdotally, based on the lucent appearance and shape of the lesions, the clinician may expect them to be more cystic in nature during surgery, however, no noted fluid was reported for tumors in this study. During gross sectioning and histologically, small cysts were noted in 6 (37.5%) cases, but soft solid tissue accounts for the majority of tumor volume.

The histomorphology of DA is often not what one would predict for a neoplasm of odontogenic epithelium. The epithelial cells of DA are polygonal, stellate, or spindle and arranged in nests and cords that generally lack tall palisading ameloblasts or stellate reticulum, but do commonly have nests and cords of epithelial cells that resemble odontogenic rests, dental lamina, and bud stage odontogenic epithelium. Furthermore, the epithelial cells often blend with a fibrous (i.e., desmoplastic) stroma that can include osteoplasia. DA can mimic both epithelial and mesenchymal tumors; therefore, it is essential that the microscopic features of the neoplasm be interpreted in the context of clinical and radiological findings.

Certain histological features can be particularly helpful for diagnosis of DA. While cell morphology is variable, it is important to note that cellular pleomorphism is not accompanied by nuclear pleomorphism. Foci of squamous epithelium are small and isolated with minimal keratinization. Inflammation and epithelial necrosis are not expected. The presence of cysts favors DA over desmoplastic carcinoma. IHC for cytokeratins can be very helpful to visualize the neoplastic epithelium within the desmoplastic background of spindle cells and collagen. Two pan-keratins were used in this study and the MNF116 gave stronger, more consistent labeling than AE1/AE3/PCK26. Relative to AE1/AE3, MNF116 has been shown to be particularly useful for poorly differentiated epithelial neoplasms (24). The osteoid matrix in DA can be surrounded by disorganized spindle cells and therefore resemble neoplastic osteoid of osteosarcoma. However, identifying odontogenic epithelium within the neoplasm is key to rule out osteosarcoma. Finally, noting the absence of aggressive invasion into adjacent soft tissue and a narrow zone of transition within bone will support a benign tumor.

Recurrence risk of DA is low in dogs treated by surgical excision. One patient (case 1) has survived over 4 years with four surgical debulking procedures. Tissue from the most recent procedure was evaluated and the histomorphology of neoplastic tissue is not significant different than tissue debulked 2 years previously. One patient (case 2) survived 34 months in remission following aggressive debulking of a tumor that been present for 3 years. In total, this Great Dane patient lived for nearly 6 years following initial tumor occurrence, dying for an unrelated reason at the age of 11 years. A third patient (case 15) had no recurrence at 2 months post-op despite incomplete histological margins. Unfortunately, this dog was lost to further follow up and eventual recurrence cannot be ruled out. This study suggests that complete excision with narrow margins may result in a surgical cure or long-term remission. Similar observations have been made for surgical treatment of humans with DA (10, 11). None of the canine patients had complete clinical staging, which is a significant limitation to this study. One patient (case 1) had slight enlargement of the ipsilateral mandibular lymph node. Although aspiration and cytological evaluation of the node were not performed, this patient remains healthy at 49 months post initial presentation.

### Differential diagnosis

Based on the clinical and radiographic features of DA, differential diagnoses to be considered include odontogenic cysts as well as other odontogenic tumors such as canine acanthomatous ameloblastoma (CAA), conventional ameloblastoma (CA), amyloid-producing ameloblastoma, and peripheral odontogenic fibroma (POF). Non-odontogenic neoplasms to differentiate include squamous cell carcinoma and to a lesser extent osteosarcoma. The latter two neoplasms are expected to progress much more rapidly compared to DA.

Similar to DA, CAA is reported to commonly affect the rostral mandible in middle aged dogs without breed or sex predilection. The conventional type of ameloblastoma commonly appears as a rostral or premolar swelling of the maxilla (7). Peripheral odontogenic fibroma has been reported to occur most commonly in the rostral maxilla (9) but is not expected to present as a lytic or mixed radiographic lesion. Nevertheless, it may be challenging to differentiate DA from other proliferative fibro-osseus lesions in both regards, clinically and radiographically.

Radiographically, CAA and CA also show bone lysis, tooth involvement, cortical bone involvement, contrast enhancement on CT, and tooth displacement (25). Regarding tooth resorption, in general, teeth at tumor sites in dogs with non-odontogenic tumors were significantly more frequently affected with external inflammatory resorption, compared with teeth at tumor sites in dogs with odontogenic tumors (26). External tooth resorption was present in 5 of 14 conventional ameloblastoma cases (7). No tooth resorption was detected in the cases of DA presented here which, may be a helpful radiographic feature for differential diagnosis.

Important histological differentials for DA in dogs are POF, odontogenic sarcoma, other proliferative fibro-osseous lesions, and carcinoma with desmoplastic stroma. Less obvious differentials are squamous odontogenic tumor, ameloblastic fibroma, and osteosarcoma. It is particularly noteworthy that 50% of the cases in this study that had had a previous biopsy were diagnosed as POF, an atypical variant of POF, or other spindle cell neoplasm. A recent study has described “hypercellular and locally invasive peripheral odontogenic fibroma”, some of which may have been DA (27). Tumors with prominent ossification may be mistaken for osteosarcoma; this same diagnostic challenge occurs with DA in humans (14).

When considered together, the clinical presentation, radiological findings, and histological features of DA are distinctive. Unlike desmoplastic carcinomas and other malignancies, the tumors in this study lacked aggressive radiological features and had mild cellular/nuclear atypia. Unlike POF, desmoplastic ameloblastoma consistently involves bone of the jaw. Unlike other fibro-osseous lesions and osteosarcoma, DA has odontogenic epithelium.

## Summary and clinical recommendations

The desmoplastic variant of ameloblastoma is an uncommon neoplasm of odontogenic epithelium in dogs, which behaves biologically like other ameloblastomas in the conventional ameloblastoma group. DA typically presents as a rostral maxillary or mandibular swelling with high likelihood of teeth being displaced. It is locally destructive with a slowly expansile growth pattern.

Advanced imaging is always advised for surgical planning since tumor borders can be ill-defined and affect several adjacent teeth. Radiologically, DA presents as a lucent or mixed lesion with well-defined borders. The recommended treatment is complete surgical excision with 5–10 mm gross surgical margin, as is considered appropriate for ameloblastoma in dogs (28). Patients in this study had a favorable prognosis following surgical excision and none had documented metastasis. Nevertheless, lack of appropriate clinical staging is a limitation of this study and further investigation is necessary to determine long-term surgical outcomes, survival, and rate of metastasis.

Veterinary pathologists will play a key role in the diagnosis of DA, which has disorganized odontogenic epithelium within a fibrous to cellular stroma and mild atypia. Careful clinicopathological correlation is essential since clinical, radiological, and microscopic features of DA overlap with benign and malignant oral tumors. This study provides information that will be useful to veterinary dentists, oral surgeons, and veterinary pathologists to improve diagnostic accuracy and treatment of dogs with ameloblastoma of the desmoplastic histological subtype.

## Data availability statement

The original contributions presented in the study are included in the article/Supplementary material, further inquiries can be directed to the corresponding author.

## Ethics statement

Ethical approval was not required for the studies involving animals in accordance with the local legislation and institutional requirements because the subjects were client-owned dogs and all treatments were provided according to best practice veterinary care. Written informed consent was obtained from the owners for the participation of their animals in this study.

## Author contributions

KF: Data curation, Formal analysis, Investigation, Methodology, Writing—original draft, Writing—review & editing. CB: Conceptualization, Data curation, Formal analysis, Investigation, Methodology, Project administration, Writing—original draft, Writing—review & editing.
